# Motivation and job satisfaction of community health workers in Ethiopia: a mixed-methods approach

**DOI:** 10.1186/s12960-023-00818-4

**Published:** 2023-05-01

**Authors:** Yohannes Ejigu, Netsanet Abera, Werissaw Haileselassie, Negalign Berhanu, Biniyam Tadesse Haile, Frehiwot Nigatu, Nurhan Tewfik, Yibeltal Kiflie, Girmay Medhin, Fasil Walelign, Mekdes Demissie, Setegn Tigabu, Daniel Taddesse, Tegene Legese Dadi, Alula Teklu

**Affiliations:** 1 Faculty of Public Health, Department of Health Policy and Management, Jimma University, Jimma, Ethiopia; 2 Monitoring, Evaluation, Research, and Quality Improvement (MERQ) PLC, Ethiopia Office, Addis Ababa, Ethiopia; 3 School of Public Health, Hawassa University, Hawassa, Ethiopia; 4 School of Public Health, Addis Ababa University, Addis Ababa, Ethiopia; 5 International Institute for Primary Health Care, Addis Ababa, Ethiopia; 6 Aklilu Lemma Institute of Pathobiology, Addis Ababa University, Addis Ababa, Ethiopia; 7 School of Public Health, Wollo University, Dessie, Ethiopia

**Keywords:** Community health workers, Health extension workers, Motivation, Job satisfaction, Primary health care, Ethiopia

## Abstract

**Background:**

Ethiopia has been providing health care to its rural population since 2004 using female Community Health Workers called Health Extension Workers (HEWs). The HEWs are credited with several achievements in improving the country's health indicators. However, information about the HEWs' motivation and job satisfaction is limited. The aim of this study was to assess the HEWs' motivation and job satisfaction, as well as the factors that influence them.

**Methods:**

A mixed-methods study was nested within a national health extension program assessment conducted from March 01 to May 31, 2019. A structured questionnaire which looked at motivation and satisfaction with Likert type single-question and multiple-item measures was used to collect quantitative data from 584 HEWs. Focus group discussion and in-depth interviews were used to gather qualitative data. Means and percentages were used to descriptively summarize important variables. Linear regression was used to identify factors associated with job satisfaction. The qualitative data was analysed thematically.

**Results:**

Overall, 48.6% of HEWs were satisfied with their job, with a mean score of 2.5 out of 4.0. The result showed a high level of satisfaction with autonomy (72%), relationships with co-workers (67%), and recognition (56%). Low level of satisfaction was linked to pay and benefits (13%), opportunities for promotion (29%), and education (34%). Regression analysis showed that HEWs in the age category of 30 years and older had lower satisfaction scores as compared to HEWs in the age category of 18–24 years (adjusted *β* = − 7.71, 95% CI: − 14.42, − 0.99). The qualitative result revealed that desire to help their community, recognition or respect gained from the community, and achievement were the major motivating factors. In contrast, inadequate pay and benefit, limited education and career advancement opportunities, workload, work environment, limited supportive supervision, and absence of opportunity to change workplace were the demotivating factors.

**Conclusions:**

The overall job satisfaction of HEWs was low; extrinsic factors, such as inadequate pay, limited education and career advancement opportunities were the major sources of demotivation. Policy makers and human resource managers should revise their human resource policies and guidelines to address the main sources of low level of job satisfaction and demotivation.

**Supplementary Information:**

The online version contains supplementary material available at 10.1186/s12960-023-00818-4.

## Introduction

Community health worker (CHW) programs have become one of the most effective strategies to address the prevailing health workforce shortages in resource-limited settings [1]. According to multiple studies, CHW programs are effective and cost-effective in delivering various health services [2-4]. A number of countries in Africa, South East Asia, and Latin America are implementing CHW programs at a national scale to meet their population’s health needs [4, 5]. However, CHW programs face performance problems and attrition of CHWs [6].

The Ethiopian government launched a national CHW program in 2004, called the Health Extension Program (HEP), with the objective of alleviating the critical shortage of health human resources and improving healthcare access to the rural population. The program uses a cadre of female CHWs called Health Extension Workers (HEWs), selected from the community among high-school graduates, trained for an additional 1 year and deployed to rural health posts. By 2018, an estimated 40,000 HEWs have been trained and deployed to health posts [7]. HEWs are expected to implement a number of prevention and treatment services categorized as disease prevention and control, family health, hygiene and environmental sanitation, and health education and communication [8]. The program has been credited for improvement of key health indicators including immunization, antenatal care, family planning, and postnatal care, among others [9-11].

Franco et al. defined motivation as “an individual’s degree of willingness to exert and maintain an effort towards organizational goals,” and job satisfaction is defined as “the extent to which a person likes his or her job.” However, motivation and job satisfaction are closely linked and influenced by similar factors at the individual, organizational, and community levels [12]. Health sector performance is critically dependent on workers’ motivation and job satisfaction [13-19]. Motivated and satisfied CHWs are expected to have good performance [20].

While motivational factors have been studied extensively among health workers in different settings, only a few studies have addressed the question of what motivates CHWs [21]. The available studies reported that interpersonal relationships at work; pride in serving the community; task sharing; hope for future employment; and gaining respect, recognition, and acceptance were identified as motivating and satisfying factors [22, 23]. On the other hand, factors such as workload, burnout, low acceptance of CHW service by the community, lack of career development, poor supervision, poor infrastructures, and inadequate pay and incentives were linked to low level of satisfaction and demotivation [24, 25].

Although the HEWs constitute a substantial proportion of health workers in Ethiopia, research evidence that helps to understand factors influencing motivation and/or job satisfaction of this workforce is limited. Existing evidence shows that pay and benefit packages, management support, supportive supervision, education opportunity, and workload influence motivation and/or job satisfaction of HEWs [23, 26]. However, these studies had a limited scope, and none of them were conducted at the national scale, justifying the need for a comprehensive national study to understand motivation and job satisfaction of HEWs. Hence, the objective of this study was to assess HEWs' motivation and job satisfaction, as well as the factors that influence them.

This study draws on Herzberg’s two-factor theory of motivations to identify factors influencing motivation and job satisfaction [27].The theory postulates that job satisfaction and dissatisfaction are not opposite ends of the same continuum. Factors that motivate individuals at work are intrinsic to work; these include achievement, responsibility, growth or advancement, recognition, autonomy, promotion and the work itself. Whereas, the factors that lead to job dissatisfaction are extrinsic (hygienic factors) and related not to the job itself but to the environment or context of the job, these include working conditions, salary, job security, company policy and administration, supervision and interpersonal relationships. Although the theory has been criticized by some scholars, it is one of the most influential theory of motivation and job satisfaction [28], and widely used in studies among health workers, including CHWs [29].

## Materials and methods

### Study area and setting

Ethiopia is a low-income country located in the horn of Africa, it has a population of an estimated 110 million, and 83% of the population are rural dwellers (agrarian and pastoralists) [30]. The country is divided into nine semi-autonomous administrative regions and two city administrations. The health system of the country follows three tiers, namely, referral hospitals, general hospitals and primary health care, where the latter includes primary hospitals, health centers and health posts. Health posts are located in rural villages and staffed with two HEWs.

### Study design

The study design was a mixed-methods cross-sectional which is nested within a national HEP assessment survey conducted from March 01 to May 31, 2019 [31]. The HEWs' motivation and job satisfaction was assessed using data gathered from HEWs working in all regions of Ethiopia.

### Sample size and sampling strategy

A single population proportion formula was used to calculate the sample size of health posts included in the study, considering a 95% confidence level and 5% margin of error (*d* = 5%) and proportion of an outcome of interest (*p* = 47%). The calculation yields 384 and the assessment then managed to include in 341 health posts and 584 HEWs working in the selected health posts. The health posts and HEWs were selected using a multi-stage cluster sampling technique. Accordingly, all districts in Ethiopia's nine regions were divided into agrarian and pastoral districts based on means of livelihood. The study districts (*n* = 67) were then selected from each stratum using a simple random. Six health posts serving the rural areas were chosen at random from each study district and included in the assessment. Finally, the satisfaction survey was conducted among 584 HEWs working at the study health posts and have a minimum of 6 month experience.

### Data collection methods

The quantitative data were collected using an interviewer administered structured questionnaire. The questionnaire included questions about demographic characteristics of HEWs and a Likert type job-related satisfaction survey. The questionnaire was adapted from a previous study conducted among nurses in Ethiopia [32], and has a reliability with Cronbach alpha coefficient of sub-scales ranges from 0.64 to 0.86. The face validity of the tool was vetted by an expert panel and it was pre-tested and necessary modifications were made based on the pre-test findings. A data collection team composed of enumerators and supervisors was employed and trained on the study objectives and procedures. In addition, field coordinators were assigned to each of the nine regions.

## Measurements

### Satisfaction and motivation

#### Satisfaction

We used 33 items and one additional overall satisfaction question to measure different domains of job satisfaction. The response of each item was recorded on an agreement scale of 1 to 4 (1 = “very dissatisfied” and 4 = “very satisfied”) and the higher scores meant a higher level of job satisfaction.

First job satisfaction was measured based on a single-question phrased as “On the whole, how do you rate your level of satisfaction with your job?” We then used eight multiple-item-based scores indicating various dimensions of job satisfaction including leadership (2 items), promotion (3 items), autonomy (4 items), work environment and cohesion (9 items), professional training opportunity (4 items), salary and benefits (4 items), recognition at work (4 items), and perceived job security (3). Third, job satisfaction was measured based on the sum total score of the 33 items as a unidimensional scale, which was verified after running exploratory factor analysis using principal component factor extraction; to examine the dimensionality of the satisfaction measuring instrument. We decided to consider the factor loading to be >  = 0.4 to be significant, the amount of variability explained by each factor to be at least 15% to be considered as a separate dimension of the instrument and eigenvalue to be above one. Having these assumptions, our data analysis resulted in four factors with eigenvalue above one. The first factor explained 64% of the total variability and the other three factors (2nd, 3rd and 4th) explained 12.9%, 6.9% and 6.3%, respectively, of the total variability. The factor loadings of 32 of the 33 items on the first factor ranged from 0.5301 to 0.7529, and the remaining one item had loading of 0.3774 (see Additional file 1). The overall Cronbach alpha value of the scale was 0.98. Hence, we considered the 33-item instrument as one-dimensional measure of ‘job satisfaction’ scale.

#### Motivation

Motivation is defined as “an individual's degree of willingness to exert and maintain an effort towards organizational goals” [12]. It is an internal psychological process and a transactional process: worker motivation is the result of the interactions between individuals and their work environment and the fit between these interactions and the broader societal context. A set of questions were asked to explore different aspects of motivation. Some of the questions include. “What motivates you to keep working as a HEW? What demotivates you to work as a HEW? What are the factors that makes your job difficult? How do you describe the level of support you get from your supervisors?”.

### Potential predictor variables

These variables include (1) sociodemographic characteristics, such as age in years, marital status (never married, married, divorced/widowed/separated) and educational attainment of HEWs, which was categorised as level three education (1 year of education after completing high school) and level four education (3 years of education after completing high school), (2) HEWs work experience in years (categorized as zero to 5 years, 6–10 years, and 11 years and above), and (3) Place of residence (categorized as ‘live in nearby town’ and ‘live in the village they work in’).

### Qualitative data collection

The HEWs’ perspectives on factors influencing their motivation and job satisfaction were also further explored through focus group discussions. In addition, experts supervising HEWs were interviewed. Altogether, we used data collected from 8 focus group discussions and 7 in-depth interviews. The topics covered include reasons for staying with the HEPs, sources of motivation, and sources of demotivation, views on monetary and non-monetary rewards, and barriers and facilitators to their work as HEWs. Experienced qualitative researchers with a minimum qualification of a master’s degree gathered the qualitative data. They were trained on the how to administer the questions, facilitate the focus group discussions and probing strategies. The discussions and interviews were audio recorded and transcribed verbatim. The quantitative and qualitative data were collected concurrently.

### Data analyses

Socio-demographic characteristics of HEWs were descriptively summarized to get an understanding of the study participants. Satisfaction was dichotomized as those who scored at and below the scale mean were considered dissatisfied and those who scored above the mean were categorized as satisfied. We generated a mean satisfaction score for each of the eight satisfaction domain and the outcome that was measured using one overall question. Weighted analysis was used after obtaining the weighting variable based on the probability of being included in the study.

The job satisfaction score computed as the sum total of responses on each of the 33 items was used as aggregate index. Then, we ran a simple linear regression to explore the association of independent variables with the job satisfaction score. The multivariable linear regression model was fitted by including variables with significant association at *p* value < 0.25 in simple linear regression. Quantitative data analyses were done in STATA 15 (StataCorp LLC, College Station, TX). Qualitative data were thematically analysed using NVivo, version 12 software. A codebook was developed based on study objectives, and relevant emergent codes were also identified based on reading of the transcripts. A team of researchers validated the codebook, and all transcribed interviews were coded. Codes were synthesized to themes related to motivating and demotivating factors.

## Results

### Background characteristics of study participants

A total of 584 HEWs were included in the study. Mean age of the respondents was 27.1 (SD: 4.2) years, and 77.7% were in the age group of 18–30 years. The majority (72.2%) of study participants were married. About half (51.6%) of the HEWs had a level three qualification (trained for 1 year), and 48.4% had a level four qualification (trained for 3 years). About 43.0% participants had more than 10 years of experience working as a HEW. The majority (71%) work in agrarian communities, while 29% serve pastoralist communities (Table 1).

**Table 1:** Background characteristics of HEWs (*N* = 584)

Background characteristics	Number of HEWs	Weighted percent
*Age category in years*		
18–24	189	22.3
25–29	272	55.4
30 and above	123	22.3
Mean age (SD)		27.1 (4.2)
*Marital status*		
Never married	159	24.2
Married	399	72.2
Others*	26	3.6
*Have children*		
Yes	369	68.1
No	215	31.9
*Education/qualification*		
Level 4 HEW	239	48.4
Level 3 HEW	345	51.6
*Working experience as HEW* (*year)*		
1–5	254	28.3
6–10	158	28.7
11–16	172	43.0
*Regions*		
Tigray	63	3.4
Afar	19	0.4
Amhara	95	20.8
Oromia	123	43.9
Somali	75	5.8
Ben-Gumuz	37	1.6
SNNP	96	23.4
Gambella	42	0.6
Harari	34	0.1
*Livelihood of communities*		
Agrarian	414	90.6
Pastoralist	170	9.4
*Place of residence of HEWs*		
Same *kebele/village* as their workplace	394	64.7
Different village/nearby town	190	35.3

*HEW* Health Extension Worker, *SD* standard deviation, *SNNPR* Southern Nations, Nationalities, and Peoples

*Others include divorced and widowed

### Level of job satisfaction

Overall, less than half (48.6%) of HEWs were satisfied with their job, with the corresponding mean score of 2.5 out of 4.0. When we look at the level of job satisfaction of each domain, about 72% of HEWs were satisfied with the autonomy, followed by the work environment, their relationships with co-workers (67%), and the recognition they get at work (56%). Only 13% of HEWs, however, were satisfied with the pay and benefit packages; 25% were satisfied with their alternative employment opportunities, and 29% were satisfied with the available opportunities for promotion. In addition, 34% were satisfied with the training/education opportunities (Table 2). The proportion of satisfied by each item and the mean satisfaction scores are presented in Additional file 2.

**Table 2:** Mean and percentage of job satisfaction by each satisfaction domain (weighted)

Satisfaction domains	Mean (SD)*	Percent satisfied
Leadership	2.52 (0.84)	44.5
Promotion	2.15 (1.00)	29.4
Autonomy	2.91 (0.81)	72.0
Work environment	2.83 (0.66)	66.9
Training opportunity	2.41 (0.75)	33.6
Pay and benefit packages	1.82 (0.81)	13.2
Recognition at work	2.74 (0.72)	56.1
Perceived job security	2.12 (0.87)	24.6
Overall job satisfaction	2.48 (0.54)	48.6

*SD* standard deviation

*The mean is calculated out of a possible score of 4

### Factors associated with HEWs’ job satisfaction

The bivariate linear regression analysis shows that a HEW’s age, marital status, experiences working as a HEW, and place of residence, as well as region and livelihood of the community served were associated with a HEW’s satisfaction at *p* value 0.25. However, only age and region remain significantly associated with HEWs’ job satisfaction in multivariable linear regression analysis. HEWs in the age category of 30 years and older had lower satisfaction scores as compared to HEWs in the age category of 18–24 years (adjusted *β* = − 7.71, 95% CI: − 14.42, − 0.99). Furthermore, there are significant differences among regions, where the HEWs served. For instance, HEWs working in the Somali region were more satisfied with their jobs as compared to those who worked in the Tigray region (adjusted *β* = 30.06, 95% CI:18.69, 41.43) (Table 3).

**Table 3:** Factors associated with job satisfaction of HEWs

Background characteristics	Number of HEWs	Mean satisfaction score	SD	Unadjusted *β* (95%CIs)	Adjusted *β* (95%CIs)
*Age category in years*					
18–24	189	85.5	19.0	1	1
25–29	272	81.6	15.4	− 3.9 (− 8.86, 1.08)	− 3.97 (− 9.01, 1.07)
30 and above	123	80.0	15.4	− 5.50 (− 11.22, 0.22)	− 7.71 (− 14.42, − 0.99)*
*Marital status*					
Never married	159	83.1	19.9	1	1
Married	399	82.1	14.9	− 0.97 (0.53, 1.11)	2.59 (− 1.73, 6.90)
Others ^a^	26	76.1	17.3	− 7.02 (− 16.63, 2.59)	− 4.42 (− 11.47, 2.62)
*Education/qualification*					
Level 3 HEW	345	81.7	18.0	1	–
Level 4 HEW	239	82.6	14.4	0.94 (− 2.74, 4.62)	–
*Work experience* (*years)*					
1–5	254	85.4	19.9	1	1
6–10	158	79.7	16.4	− 5.62 (− 11.04, − 0.20)*	− 0.67 (− 5.58, 4.24)
11–16	172	81.7	13.2	− 3.68 (− 8.38, 1.10)	3.51 (− 1.79, 8.82)
*Region*					
Tigray	63	81.5	11.6	1	1
Afar	19	82.6	10.7	1.04 (− 4.81, 6.89)	3.49 (− 4.89, 11.86)
Amhara	95	79.8	14.0	− 1.76 (− 6.01, 2.48)	− 2.73 (− 7.27, 1.82)
Oromia	123	78.8	13.0	− 2.70 (− 6.97, 1.58)	− 4.49 (− 9.42, 0.43)
Somali	75	109.5	29.7	28.00 (18.02, 37.98)	**30.06 (18.69, 41.43)**
Ben-Gumuz	37	91.9	13.3	10.37 (4.73, 16.02)	**9.07 (2.62, 15.52)**
SNNP	96	83.3	13.4	1.78 (− 2.79, 6.35)	1.27− (3.32, 5.85)
Gambella	42	75.1	20.9	− 6.41 (− 14.93, 2.11)	− 6.56 (− 15.22, 2.11)
Harari	34	86.8	11.2	5.28 (0.25, 10.31)	3.97 (− 1.55, 9.48)
*Livelihood of communities*					
Agrarian	414	80.5	13.5	1	1
Pastoralist	170	97.8	28.6	17.24 (10.49, 23.99)	2.48 (− 7.02, 2.06
*Place of residence of HEWs*					
Same as workplace	394	82.9	17.5	1	-
Other village or nearby town	190	80.8	13.9	2.10 (− 5.90, 1.70)	–

The mean is calculated out of the total 132 possible score

*HEWs* Health Extension Workers, *SNNP* Southern Nations, Nationalities, and Peoples, *SD* standard deviation

*Others include divorced, widowed, and separated

### Qualitative results

In addition to the quantitative data, qualitative data were collected through open-ended in-depth-interviews and focus group discussions to identify the factors that motivate and demotivate the HEWs. The major motivating and demotivating factors identified through qualitative exploration are summarized in Table 4.

**Table 4:** Major motivating and demotivating factors identified by the qualitative study

Motivating factors	Demotivating factors
Desire to help the community	Inadequate pay and benefits
Recognition by the community	Lack/limited career advancement/promotion
Achievement	Limited education opportunity
Skills acquired (communication skill)	Workload and work environment
Peer cohesion/support	Limited support from top management
	Lack of transfer opportunity
	Community resistance to change

### Motivating factors

The HEWs reported that the main motivators for themselves were to help their community and contribute to good outcomes, such as reduced maternal and child mortality, and better sanitation and hygiene indices. In addition, community members' recognition, acceptance, and respect were identified as sources of motivation along with the work environment and peer support (relationships with other HEWs). HEWs also stated that they developed good communication skills that enable them to provide community health education and understand community needs. Possessing of such communication skills boosted their confidence and motivation to continue working as a HEW. For example, two of the sampled HEWs said the following while describing the various factors that motivate them in their work.

“First of all, we are helping our fathers, mothers, brothers, and sisters to adopt healthy behavior. Helping them changing their behavior is very important. For instance, observing a reduction in mother and child death gives me great satisfaction.”

HEW working in agrarian community

“I am so happy working as an HEW because I have a mother, and working for mothers, convincing them to utilize health services makes me happy. Assisting mothers to deliver in health facility is an honor. Besides, working in my village, when the community members ask me for support, believing in me gives me great satisfaction.”

HEW working in agrarian community

### Demotivating factors

HEWs also noted a number of demotivating factors, including limited/lack of opportunity for career advancement, promotion, and education opportunities and inadequate pay and benefit packages. In addition, inadequate support from management, poor infrastructure, and workload were mentioned as sources of demotivation. Some HEWs were also tasked by their supervisors to do activities that are not related to health, including collecting taxes and health insurance premiums.

While discussing the demotivating factors in their work, HEWs have said the following:

“I feel disappointed that when you work as HEW there is no career advancement. It has been thirteen years since the Health Extension Program started, and the HEWs who were hired back then, there is no evaluation of their performance. No educational opportunities of any sort. There is no incentive, no change at all. When I think about this, I feel disappointed.

HEW working in pastoralist community

“The work is really hard. Even the community named us “burned faces!” because of the toughness of the job. We are sacrificing for our country. We go from home to home to give services, walking for three hours, but no incentive for it.”

HEW working in agrarian community

“Our salary does not match our work; the work is hard. The education opportunity that has begun recently does not satisfy our education needs.”

HEW working in pastoralist community

“If you look at their work, they deserve great respect, but their salary is not adequate.…we also feel that it is unfair to complain about their performance, given low salary…”

Health office head, from Southern Ethiopia

Although the overall community acceptance of the works of HEWs is very high, some community members are not appreciative of HEWs’ work or resist change, which is mentioned as a demotivating factor. In describing the resistance of some community members, HEWs stated their personal experiences as follows:

“We conduct home visits to provide health education. I remember once the woman (homeowner) yelled at me to get out of her house. I felt ashamed, and I don’t forget that moment.”

An HEW working in agrarian community

“…. I feel ashamed and question myself when the community resists change… I sometimes become hopeless….”

An HEW working in agrarian community

Because HEWs are expected to serve the community in which they were born, their desires to change their work location have been largely ignored. Prevention of HEWs from transferring to other workplaces was frequently mentioned as a major source of demotivation. In explaining their disappointment, HEWs working in agrarian communities were quoted as follows:

“…I am working in a village where I was born. The problem is that my husband lives in another area, and when I ask for transfer, it is impossible.”

HEW working in agrarian community

“…Even if the Health Extension Program is good for the community, it is creating a problem for the HEWs, because we can’t change workplace, or transfer from one place to other.”

HEW working in agrarian community

## Discussion

In this study, the motivation and job satisfaction of HEWs in rural Ethiopia were investigated. Only 48.6% of HEWs were satisfied with their jobs. From the job satisfaction domains evaluated, HEWs were most satisfied with their autonomy, the work environment, their relationships with coworkers, and the recognition they get at work. On the other hand, they were the most unsatisfied with their pay and benefits packages, with only 13% reporting satisfaction. The qualitative findings showed that the desire to help the community, community recognition or respect, and job performance were identified as key motivating factors. Whereas, inadequate compensation and benefit packages, restricted education and professional advancement or prospects, workload, work atmosphere, and limited support from supervisors were the demotivating factors.

Studies showed that motivated and satisfied workers have good performance, and the opposite is true if workers are not satisfied or demotivated [20]. Hence, the finding of a low level of satisfaction among HEWs may negatively affect their performance. In fact, the level of job satisfaction among HEWs in the current study was higher than what was reported in small studies in Southern and Central Ethiopia [26, 33], but lower than that of a study done in four Ethiopian regions [23]. The inconsistency seems to be due to methodological differences between the different studies and small sample size used by previous studies.

HEWs are government employees and have regular salary and benefit packages, like other health workers in the public sector; however, this study showed that HEWs were less satisfied with their pay and benefit packages. The finding is consistent with the previous studies from Ethiopia reporting low level of satisfaction of HEWs with the pay and benefit packages [26, 33] and from other studies of resource-limited settings [34, 35]. In fact, most researches focusing on job satisfaction and motivation of any category of health workers in resource-limited settings reported that workers are less satisfied due to insufficient pay [29, 36-38]. Studies also demonstrated that unpaid volunteer CHWs were found to be worse off than their peers in various psychosocial and economic respects, reinforcing the recommendation that CHWs in low-income settings be paid [39, 40]. Maslow's hierarchy of needs theory and Herzberg's two factor or motivator-hygiene theory both support the importance of low pay in demotivating workers [27, 41]. In addition to, or instead of, financial incentives, some research has suggested that intrinsic motivators play a role among CHWs [29, 42-45].

The qualitative results of motivating and demotivating factors also reiterate the importance of intrinsic needs and values such as a desire to help their community, gain recognition or respect of the community, and find achievement as the major motivating factors. In contrast, hygienic factors such as inadequate pay and benefit packages, limited education and career advancement opportunities, high workload, limited supportive supervision, and absence of opportunity to change workplace were the demotivating factors. It appears that the finding seems largely in line with Herzberg’s motivator-hygiene theory, which states that motivating factors are wholly different from demotivating factors. Motivator factors are usually intrinsic and determine job satisfaction and motivation, whereas hygienic factors determine demotivation and tend to be extrinsic, related to the environment [27]. Similar findings were also documented by a number of studies [23, 29, 43].

The regression analysis showed that HEW’s age was inversely associated with job satisfaction. This might be because the HEWs’ jobs require frequent travel on foot to conduct home visits and give health education, and this type of job may not be as suitable for older people. Notably, previous studies did not report an association between age and job satisfaction [23, 26]. The current study also revealed that HEWs from some regions (eg. Somali and Ben-Gumuz) have significantly higher level of job satisfaction. The disparities in HEWs’ job satisfaction across regions might be due to differences in the amount of pay to HEWs. At the time of this study, the pay and benefit packages were widely different across regions.

This study should be understood in light of the following strengths and limitations. The study is based on population data collected from all over the country with a nationally representative sample of HEWs. To our knowledge, this is the first national-scale study addressing the question of HEWs’ motivation and job satisfaction. However, the following possible limitations should be highlighted. Although respondents were assured about the confidentiality of their responses, it is possible that they were hesitant to express their true feelings about questions related to challenges of management support and supervision for fear of the authorities. Although, the face validity of the tool was assessed by a panel of experts, its content and construct validity was not tested.

## Conclusion

In conclusion, the level of job satisfaction and motivation among rural HEWs was low. Inadequate pay and benefits packages, job security, and promotion and training opportunities were the major factors of low satisfaction. Moreover, the qualitative study has identified inadequate pay and benefit packages, limited education and career advancement opportunities, workload, work environment, limited supportive supervision, and absence of opportunity to change workplace as the major demotivating factors. Policy makers and human resource managers working at Ministry of Health of Ethiopia and regional offices should revise their human resource policies and guidelines to address HEWs’ demotivation and low level of satisfaction, including developing a clear career development path and education opportunities for HEWs. It is also imperative for regional and district level managers to amend their policies, so that HEWs can change job location. District level managers should conduct a regular supportive supervision and consider recruiting additional HEWs in areas, where there is a heavy workload.

## Supplementary Information

Additional file 1: Table S1. Factor loadings.

Additional file 2: Table S2. Mean and percentage of job satisfaction by each item.

## Data Availability

Data can be available upon request.

## Abbreviations

CHW: Community health worker
FGD: Focus group discussion
HEW: Health extension workers
HEP: Health Extension Program
