# Supplementary material for: Motivation and job satisfaction of community health workers in Ethiopia: a mixed-methods approach
Source: Hum Resour Health. 2023 May 1;21:35. doi: 10.1186/s12960-023-00818-4 (PMC10152586; doi:10.1186/s12960-023-00818-4)
Supplement: Supplementary file 1 — Additional file 1: Table S1. Factor loadings. [file 12960_2023_818_MOESM1_ESM.docx]

| **Variables** | **Factor 1** |
| --- | --- |
| Level of administration support | 0.5370 |
| Recognition of your work by your supervisor | 0.5700 |
| Support for continuing education | 0.6871 |
| Opportunity for professional growth | 0.7183 |
| Personal growth and development through education and training | 0.7360 |
| Autonomy to make work related decisions | 0.6906 |
| Autonomy to be fully accountable for your own decisions | 0.7172 |
| Opportunity to work alone on the job | 0.6600 |
| Freedom to apply your own judgment on job | 0.7167 |
| The working environment allows you to make autonomous work related decisions | 0.7529 |
| The working environment allows you to be accountable for your own decisions | 0.7044 |
| The working environment encourages you to make change in your practice to suit the community needs | 0.7118 |
| The working environment to provide a stimulating intellectual environment. | 0.7402 |
| The working environment to enable you to demonstrate a high level of competence | 0.7353 |
| The working environment gives you an opportunity to expand your scope of practice | 0.7103 |
| The relationship among staff in your work place | 0.5377 |
| Group members positively influences one another | 0.5668 |
| The relationship with colleagues in your facility | 0.5301 |
| Training opportunities available to you | 0.6541 |
| Training program appropriateness to enhance your job performance | 0.5893 |
| Availability of training and orientation to new staff | 0.6283 |
| Opportunity to participate in research | 0.5985 |
| Salary and compensation packages | 0.6184 |
| Employment benefit packages | 0.6522 |
| Amount of pay in relation to what the cost of living in this area | 0.6125 |
| The adequacy of the of compensation for the work you do | 0.3774 |
| Sense of value for what you do | 0.5508 |
| Consideration given to your personal needs | 0.6316 |
| Consideration given to your opinion and suggestion by your supervisors | 0.6199 |
| Recognition of your work by peers | 0.5377 |
| Job security or presence of other opportunity if you quit your current job | 0.5540 |
| The opportunity to attain a suitable position in same or other organizations | 0.5933 |
| It would be easy to find acceptable alternative job | 0.5823 |

Supplementary table 1. Factor loadings
