# Supplementary material for: Motivation and job satisfaction of community health workers in Ethiopia: a mixed-methods approach
Source: Hum Resour Health. 2023 May 1;21:35. doi: 10.1186/s12960-023-00818-4 (PMC10152586; doi:10.1186/s12960-023-00818-4)
Supplement: Supplementary file 2 — Additional file 2: Table S2. Mean and percentage of job satisfaction by each item. [file 12960_2023_818_MOESM2_ESM.docx]

# Supplementary table 2.Mean and percentage of job satisfaction by each item

| **Job satisfaction statement** | **Level of satisfaction (%)** | | | | **Mean satisfaction score** | |
| --- | --- | --- | --- | --- | --- | --- |
|  | **Very Dissatisfied** | **Moderately Dissatisfied** | **Moderately Satisfied** | **Very Satisfied** | **Mean** | **Standard deviation** |
| Level of administration support | 14.8 | 23.9 | 45.4 | 15.8 | 2.62 | 0.92 |
| Recognition of your work by your supervisor | 11.2 | 22.9 | 45.7 | 20.2 | 2.75 | 0.90 |
| Support for continuing education | 29.6 | 25.7 | 28.2 | 16.5 | 2.32 | 1.07 |
| Opportunity for professional growth | 30.8 | 26.0 | 28.4 | 14.9 | 2.27 | 1.06 |
| Personal growth and development through education and training | 26.8 | 27.8 | 30.9 | 14.5 | 2.33 | 1.02 |
| Autonomy to make work related decisions | 8.9 | 18.0 | 47.5 | 25.6 | 2.90 | 0.88 |
| Autonomy to be fully accountable for your own decisions | 6.7 | 19.1 | 48.5 | 25.8 | 2.93 | 0.84 |
| Opportunity to work alone on the job | 5.3 | 19.1 | 49.6 | 26.0 | 2.96 | 0.81 |
| Freedom to apply your own judgment on job | 6.5 | 17.2 | 50.7 | 25.6 | 2.95 | 0.83 |
| The working environment allows you to make autonomous work related decisions | 8.0 | 25.9 | 45.2 | 20.9 | 2.79 | 0.86 |
| The working environment allows you to be accountable for your own decisions | 7.7 | 25.0 | 43.8 | 23.5 | 2.83 | 0.87 |
| The working environment encourages you to make change in your practice to suit the community needs | 8.7 | 24.5 | 48.0 | 18.7 | 2.77 | 0.85 |
| The working environment to provide a stimulating intellectual environment. | 11.5 | 32.0 | 39.7 | 16.8 | 2.62 | 0.90 |
| The working environment to enable you to demonstrate a high level of competence | 11.0 | 30.1 | 41.6 | 17.3 | 2.65 | 0.89 |
| The working environment gives you an opportunity to expand your scope of practice | 10.8 | 30.8 | 42.8 | 15.6 | 2.63 | 0.87 |
| The relationship among staff in your work place | 3.9 | 10.9 | 43.9 | 41.1 | 3.23 | 0.79 |
| Group members positively influences one another | 4.2 | 12.3 | 47.4 | 36.0 | 3.16 | 0.80 |
| The relationship with colleagues in your facility | 3.2 | 8.8 | 45.9 | 42.0 | 3.27 | 0.75 |
| Training opportunities available to you | 16.5 | 28.4 | 38.2 | 16.9 | 2.55 | 0.96 |
| Training program appropriateness to enhance your job performance | 9.1 | 21.6 | 41.4 | 27.9 | 2.88 | 0.92 |
| Availability of training and orientation to new staff | 13.5 | 31.0 | 38.4 | 17.1 | 2.59 | 0.92 |
| Opportunity to participate in research | 39.1 | 28.1 | 20.3 | 12.5 | 2.06 | 1.04 |
| Salary and compensation packages | 52.2 | 25.2 | 12.5 | 10.1 | 1.80 | 1.01 |
| Employment benefit packages | 44.8 | 29.9 | 15.3 | 10.0 | 1.90 | 1.00 |
| Amount of pay in relation to what the cost of living in this area | 51.5 | 28.0 | 11.7 | 8.9 | 1.78 | 0.97 |
| The adequacy of the of compensation for the work you do | 32.9 | 21.0 | 14.6 | 31.5 | 2.45 | 1.24 |
| Sense of value for what you do | 8.4 | 20.8 | 45.3 | 25.6 | 2.88 | 0.89 |
| Consideration given to your personal needs | 15.8 | 31.6 | 37.6 | 14.9 | 2.52 | 0.93 |
| Consideration given to your opinion and suggestion by your supervisors | 10.8 | 29.3 | 41.2 | 18.7 | 2.68 | 0.90 |
| Recognition of your work by peers | 6.9 | 16.7 | 51.0 | 25.3 | 2.95 | 0.83 |
| Job security or presence of other opportunity if you quit your current job | 25.8 | 35.3 | 27.0 | 11.9 | 2.25 | 0.97 |
| The opportunity to attain a suitable position in same or other organizations | 28.4 | 34.5 | 25.1 | 12.0 | 2.21 | 0.99 |
| It would be easy to find acceptable alternative job | 29.7 | 35.2 | 24.1 | 11.0 | 2.16 | 0.98 |
